# Supplementary material for: BtuB TonB-dependent transporters and BtuG surface lipoproteins form stable complexes for vitamin B12 uptake in gut Bacteroides
Source: Nat Commun. 2023 Aug 5;14:4714. doi: 10.1038/s41467-023-40427-2 (PMC10404256; doi:10.1038/s41467-023-40427-2)
Supplement: Supplementary file 1 — Supplementary information [file 41467_2023_40427_MOESM1_ESM.pdf]

Supplementary Table 1 | X-ray crystallographic data collection and refinement statistics. Values in parentheses are for the highest resolution shell

	BtuG2-CNCbl Co-SAD	BtuG2-CNCbl hi-res	BtuG2-AdoCbl	BtuG2-Cbi	BtuB2G2	BtuG3-CNCbl
**Data collection**						
DLS beamline	i24	i24	i03	i24	i04	i03
Wavelength	1.54987	0.96858	0.89843	0.99987	0.97950	0.9794
Space Group	I222	$P2_1$	$P2_1$	$P4_12_12$	C2	$P2_12_12_1$
Cell dimensions						
a,b,c (Å)	59, 129, 141	80, 59, 101	80, 58, 101	79, 79, 155	280, 154, 107	74, 131, 179
α,β,γ (°)	90, 90, 90	90, 103, 90	90, 104, 90	90, 90, 90	90, 106, 90	90, 90, 90
Molecules in AU	1	2	2	1	3:3	4
Resolution range (Å)	64.6-1.9 (2.0-1.9)	59.1-1.7 (1.7-1.7)	70.3-2.3 (2.4-2.3)	43.30-1.33 (1.36-1.33)	76.3-3.7 (3.8-3.7)	74.7-2.6 (2.68-2.60)
I/ σI	12.2 (1.3)	6 (1.4)	5.1 (1.5)	16.7 (0.9)	4.2 (1)	11.8 (1.1)
Completeness (%)	99.9 (99.9)	99.4 (96.8)	100 (100)	99.9 (98.3)	99.3 (99.8)	99.4 (99)
Redundancy	32.5 (26.7)	3.5 (3.6)	6.8 (6.5)	24.2 (17.9)	3.8 (3.9)	13.2 (13.6)
Rpim (%)	4 (53.1)	7 (51)	10 (44)	2.1 (82)	16 (85)	6 (96)
CC (1/2)	1 (0.69)	0.98 (0.56)	0.98 (0.69)	1 (0.3)	0.9 (0.3)	1 (0.6)
Anomalous completeness	99.8 (99.8)	-	-	-	-	-
Anomalous redundancy	15.9 (12.9)	-	-	-	-	-
**Phasing**						
SOLVE FOM	0.25	-	-	-	-	-
Sites found [expected]	1 [1]	-	-	-	-	-
**Refinement**						
Resolution (Å)	-	59.1-1.72	70.2-2.3	43.3-1.33	67.02-3.72	74.0-2.6
Rwork/Rfree (%)	-	17.9/22.3	22.3/26.8	14.2/16.8	23.9/29.1	0.21/0.27
Reflections	-	97918	41442	113069	45634	54623
No. Atoms	-					
Protein	-	5489	5454	2817	23359	10619
Corrinoid	-	186	218	68	-	372
B-factors (Å2)	-					
Protein	-	20.3	36.3	23.4	123.8	74.1
Corrinoid	-	16.8	27.5	19.6	-	74.2
Rmsd	-					
Bond lengths (Å)	-	0.007	0.012	0.012	0.004	0.014
Bond Angles (°)	-	1.7	1.8	1.2	1.07	2.4
Molprobity clashscore	-	2.4	4	1.1	13.5	5.3
Ramachandran plot	-					
Favoured (%)	-	93.7	92.3	93.2	91.2	92
Disallowed (%)	-	0	0.9	0.3	0.49	1.2
PDB code	-	8BMX	8BMY	8BMZ	8BM0	8OKV

Supplementary Table 2 | Cryo-EM data collection, image processing and refinement statistics.

	BtuB1G1	BtuB3G3-CNCbl state 1	BtuB3G3-CNCbl state 2
**Data collection**			
Electron microscope	FEI Titan Krios	FEI Titan Krios	
Voltage (kV)	300	300	
Spherical aberration (μm)	2.7	2.7	
Camera	Falcon 4 (counting)	Falcon 4i (counting)	
Energy filter	Selectris X (5 eV slit)	Selectris X (10 eV slit)	
Magnification	130,000	165,000	
Pixel size (Å)	0.91	0.74	
Total dose (e^-/Å^2)	35	35.6	
Dose rate (e^-/pixel/s)	6.22	5.95	
Defocus minimum maximum (μm)	-0.9 to -2.4	-0.8 to -2.3	
Number of EPU frames	160	1008	
Number of movies collected	1,924	9,826	
**Image Processing**			
Initial number of particles	1,110,283	3,311,038	
Final number of particles	50,547	66,318	91,067
Global resolution (FSC = 0.143)	3.22	2.97	2.75
Map sharpening	B-factor (–50 Å^2)	B-factor (–38.6 Å^2)	B-factor (–47.5 Å^2)
**Refinement**			
Model composition			
Non-hydrogen atoms	9883	7830	7837
Protein residues	1250	956	958
R.m.s. deviations			
Bonds lengths (Å)	0.005	0.003	0.004
Bond angles (˚)	1.044	0.557	0.612
Validation			
Molprobity score	1.91	1.93	1.96
Clash score	6.97	9.23	8.24
Rotamer outliers (%)	0	0	
Ramachandran plot			
Favoured (%)	90.71	93.2	91.16
Outliers (%)	0	0.3	0.3
PDB	8BLW	8P98	8P97
EMDB	EMD-16114	EMD-17575	EMD-17574

Supplementary Table 3 | Oligonucleotides used in this study.

**Cloning BT1954 in pET28**	
BT1954FNcoI	CTAGCCATGGGCAAATGGGATTACGGAGAGATGGAAGATT
BT1954RXhoI	CTAGCTCGAGTTTCCAGCAGAAAGCTCCCGGAA
**Cloning BT2095 in pET28**	
2095NtHisNcoI	CAGTCCATGGGCCACCATCACCATCACCATCCTTTCAGGGCTACAGGAGATGG
2095NtXhoI	CAGTCTCGAGTTACTTCCAACAAAATGCTCCG
**Introducing His tag in BT1953**	
BT1953-1000UF	CTAGGTCGACGCTGTTCACGGCCGGTGT
BT1953-UR	TTAGTGATGGTGATGGTGATGTCGTTTACTATTTTTGTTTTTTCCGAACTTGGG
BT1953-DF	AAAAATAGTAAACGACATCACCATCACCATCACTAATCTGCATCAATATGAACG CATTAAAGAATTTAAGC
BT1953-1000DR	CTAGTCTAGATACACCTCTCCATTTTTGAAACATCGG
**Replacing wt promoter of Locus 1 with P1E6**	
L1UF	CGCAGAATCAGGAGTTTCAATTACTG
L1UR	TGAGCCTTTCGTTTTATCTTAAAAAGCTTTGAATCCAACCACC
P1E6L1F	GCTTTTTAAGATAAAACGAAAGGCTCAGTCGAAAG
P1E6L1R	GGAATGTTATTTTTTAAATGTTGTGTG
L1DF	CTGATCACACAACATTTAAAAAATAACATTCCATGAGAAGGAATACTTTTATTAAAAA GATGAGC
L1DR	GGACAGGTAATATTCTTGCTGGTA
Locus1finalsphISalI	ACGCGCATGCGTCGACCCAACAAAGCGGAGAACTCTATG
Locus1finalXbaI	ACGCTCTAGACCTCCGACGGAGGTAAGAGCATC
**Introducing His tag in BT1489**	
BT1489-UFSal	ACGCGTCGACTACCAGCAAGAATATTACCTGTCC
BT1489-UtagR	CTAATGGTGATGGTGATGGTGTCCACCTCCATACCTGACTCCTATCGTCACTC
BT1489-DtagF	GGAGGTGGACACCATCACCATCACCATTAGGTAATAGGTATTAAGTAATAAGTATTAGG
BT1489-DRXba	ACGCTCTAGAATTCAGTTGAATGCTGAAAGGCG
**Replacing wt promoter of Locus 3 with P1E6**	
L3UF	CCACTCCGTACCAGTATGGCTGTG
L3UD	TGAGCCTTTCGTTTTATCTTATAGTGAATTACGAATATTTAAATTC
P1E6L3F	TTCACTATAAGATAAAACGAAAGGCTCAGTCGAAAG
P1E6L3R	GGAATGTTATTTTTTAAATGTTGTGTG
L3DF	CACACAACATTTAAAAAATAACATTCCATGAAACGAATTTTACTTTCTG
L3DR	CCTCCGTATAGCGATAAATCATCC
L3FinsphISalI	ACGCGCATGCGTCGACGTTGGTAGTTGATTATGTGATCG
L3FinXbaI	ACGCTCTAGAGGATGATTTATCGCTATACGGAGG
**Introducing His tag in BT2094**	
2094FSal	ACGCGTCGACCTTTCTACGGGCAGTTGGATGGAG
2094Rtag	CTAATGGTGATGGTGATGGTGTCCACCTCCTTTTTTCTTCTTGCCCCACTTGGGAG
2094Ftag	GGAGGTGGACACCATCACCATCACCATTAGGTGGGTGATAGCCGGACTCCTGTGC
2094RXba	ACGCTCTAGAGGTGACATCCAGTGATGCACCGGC
SaldelE8-B3	ACGCGTCGACTATACTGATTCTAACCGTGAAC
XbadelE8-B3	ACGCTCTAGAGTCGATTCCCAAACGGGCAGCC
**Replacing EL8 from BT2094 with Gly-Gly**	
Q5delE8-B3F	GGTCCCGTTAATCTTAAAAAAGTAC
Q5delE8-B3R	TCCCCAAATAATCCAGTCGTTTATATG

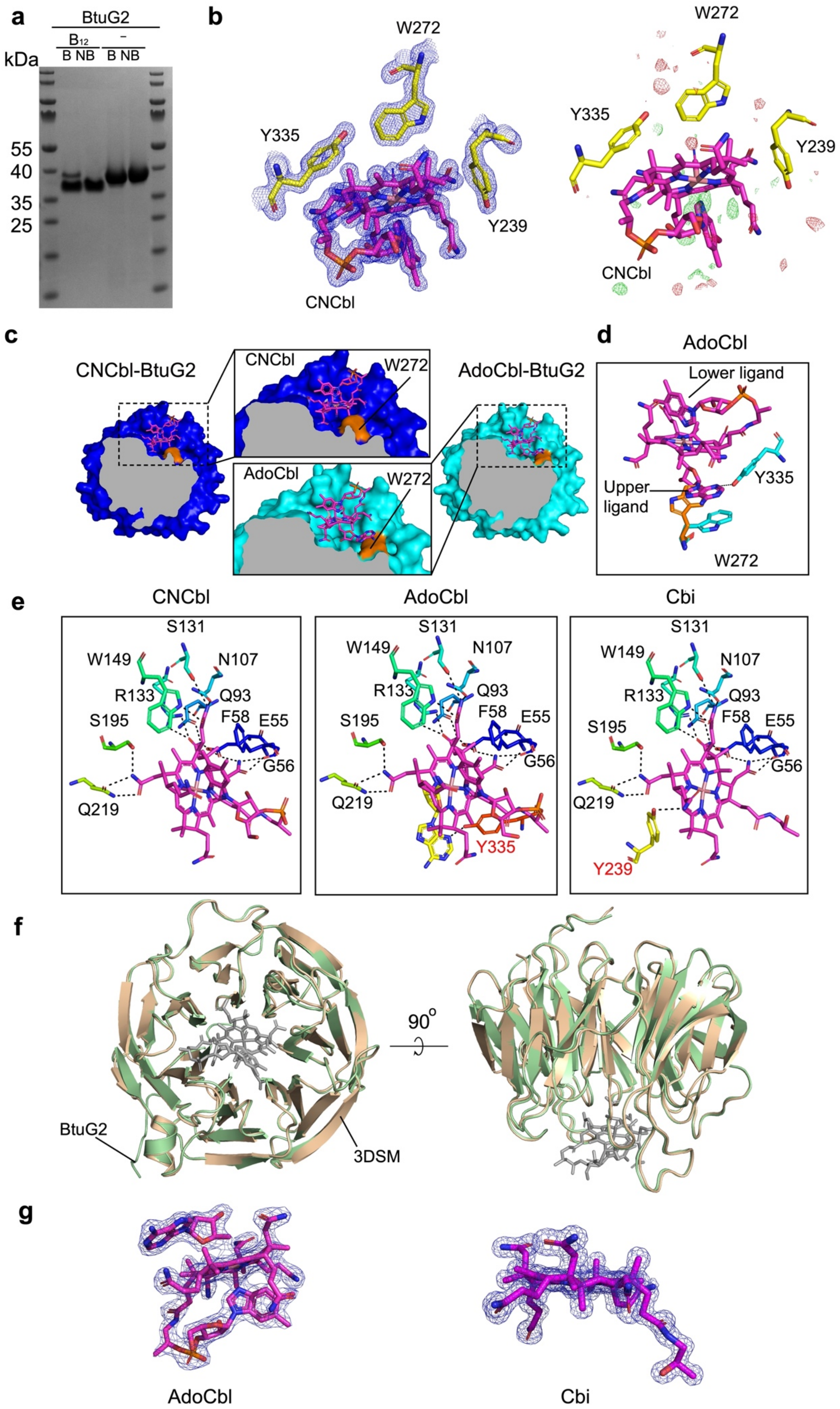
a
BtuG2
B12
–
B NB
B NB
kDa
55
40
35
25
b
W272
Y335
Y239
CNCbl
W272
Y335
Y239
CNCbl
c
CNCbl-BtuG2
CNCbl
W272
AdoCbl
W272
AdoCbl-BtuG2
d
AdoCbl
Lower ligand
Upper ligand
Y335
W272
e
CNCbl
S131
W149
N107
R133
Q93
S195
F58
E55
G56
Q219
AdoCbl
S131
W149
N107
R133
Q93
S195
F58
E55
G56
Q219
Y335
Cbi
S131
W149
N107
R133
Q93
S195
F58
E55
G56
Q219
Y239
f
90°
BtuG2
3DSM
g
AdoCbl
Cbi

Supplementary Figure 1 l BtuG2 binds different corrinoids. **a**, Representative SDS-PAGE gel (n = 3 independent experiments) showing the different mobility of BtuG2 bound to CNCbl (B_{12}). B stands for boiled, NB for non-boiled. Note the double band on the CNCbl-BtuG2 boiled sample; the higher band corresponds to apo-BtuG2 after B_{12} removal due to the boiling. Uncropped gels are in Source Data. **b**, Figure showing a stick representation of CNCbl bound to BtuG2 with the 2Fo-Fc electron density at 1.5 σ (left panel) and Fo-FC map at 3 σ (right panel). For reference, three residues from BtuG2 are shown in yellow. **c**, Slice of a surface representation for CNCbl-BtuG2 (blue) and AdoCbl-BtuG2. Close up views to show the cavity in which the upper ligand (-CN or -5'deoxydenosyl respectively) is located. W272 is depicted in orange; this residue is displaced in AdoCbl-BtuG2, enlarging the binding pocket. **d**, Residues implicated in the upper ligand stabilization of AdoCbl (coloured in cyan). The orange W272 corresponds to the position of the side chain in the CNCbl-BtuG2 structure. **e**, Panels displaying the residues involved in hydrogen bonding between BtuG2 and different corrinoids seen in the crystal structures. Common residues for the binding of the three corrinoids have black labels, those implicated in interacting with only one of the corrinoids red. Residues in rainbow colouring are ramped from blue at the N terminus to red at the C terminus. Hydrogen bonds are represented as black dashed lines. In middle panel the upper ligand, 5'-deoxy-5'-adenosyl, is coloured in yellow. **f,** Superposition of 3DSM, in wheat (apo BtuG2), and BtuG2-CNCbl (protein in pale green and CNCbl in grey). Notice that the structures are virtually the same regardless of the presence of the ligand (Cα RMSD 0.43 Å). **g**, Figure showing a stick representation of AdoCbl (right panel) and Cbi (left panel) bound to BtuG2 with the 2Fo-Fc electron density contoured at 1.5 σ.

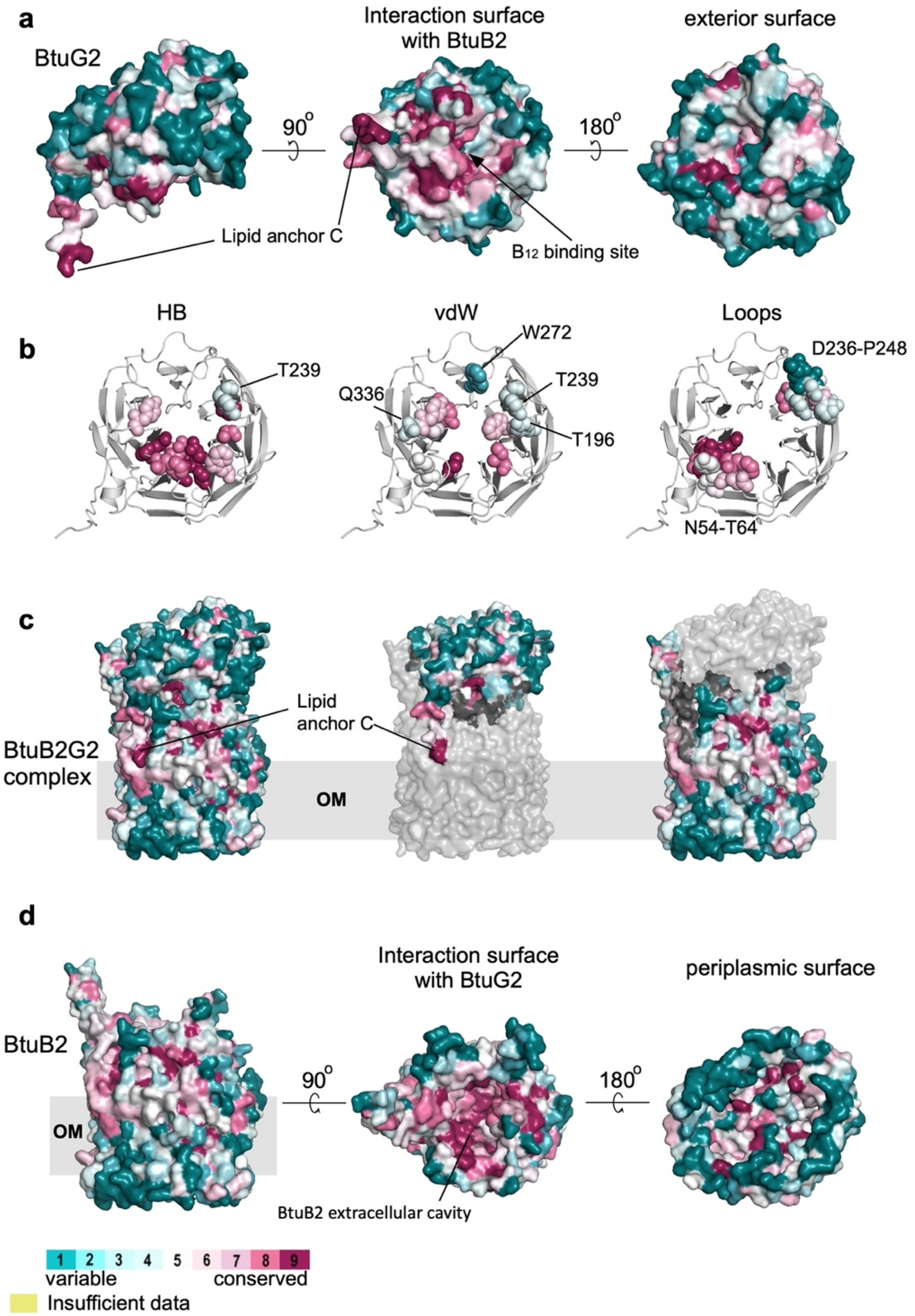

Supplementary Figure 2 l ConSurf analyses of BtuG2 and BtuB2. **a,** Several views of the surface representation of BtuG2 coloured with the conservation scores. **b,** Cartoon representation of BtuG2 showing the level of conservation of residues implicated in hydrogen Bonding (left panel), van der Waals forces (middle panel), and interacting loops derived from the structural and molecular dynamic simulations (right panel) implicated in ligand binding. **c,** Surface representation of BtuB2-BtuG2 complex with conservation score colouring. In the middle panel BtuB2 is in grey and in the right panel BtuG2 is grey. **d,** Similar to (a), but for BtuB2.

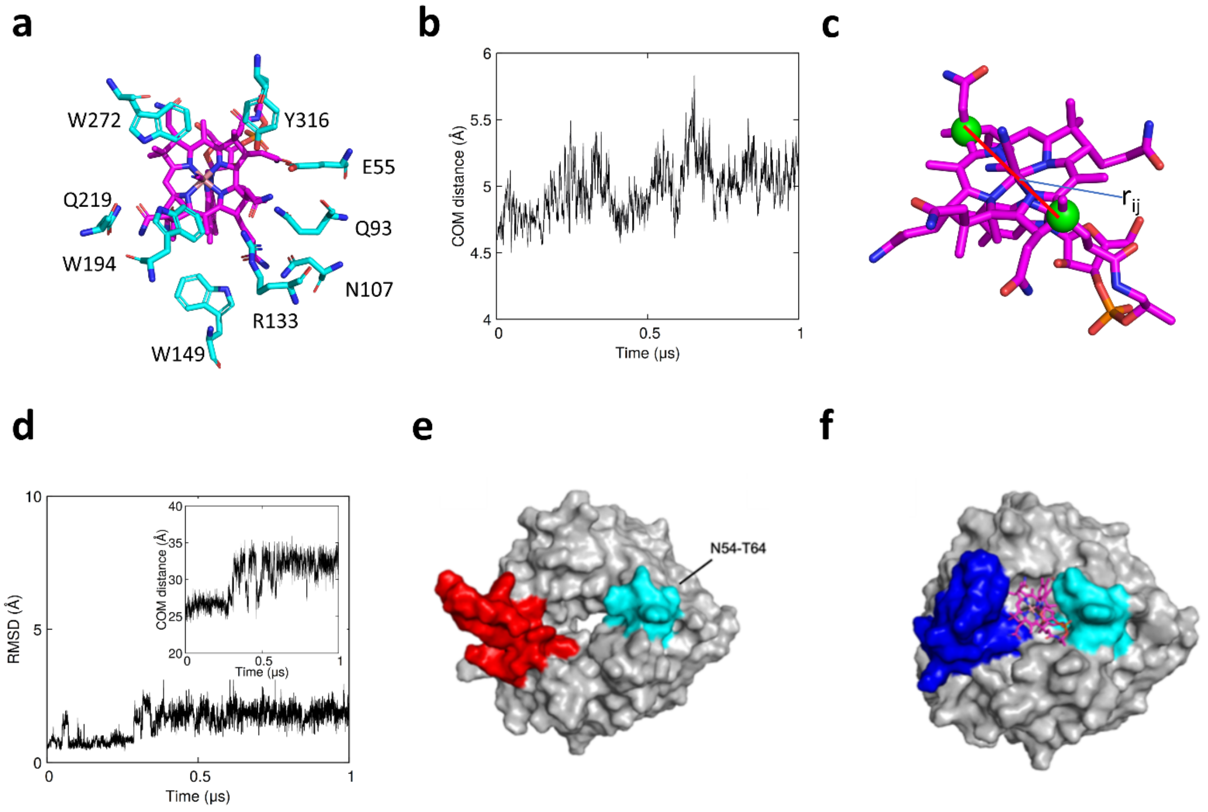

Supplementary Figure 3 l Analysis of CNCbl binding to BtuG2 using MD simulations. a, CNCbl together with the BtuG2 amino acid residues that are within 3 Å COM distance (based on the crystal structure). **b,** COM distance between the BtuG2 binding pocket and CNCbl for simulations starting from the crystal structure. **c,** The projection of the vector r_{ij} between the carbon atoms depicted as green spheres onto the z axis is defined as collective variable z_{ij}. **d**, RMSD values of loop β5A (D236-P249) and COM distance between BtuG2 protein and loop β5A (inset), depicting the relaxation of BtuG2 protein in the absence of CNCbl. **e-f**, Surface representation showing loops β5A and N54-T64 from the relaxed state (left panel; β5A red) and crystal structure (right panel; β5A blue). The loop N54-T64 (β1A) is coloured cyan.

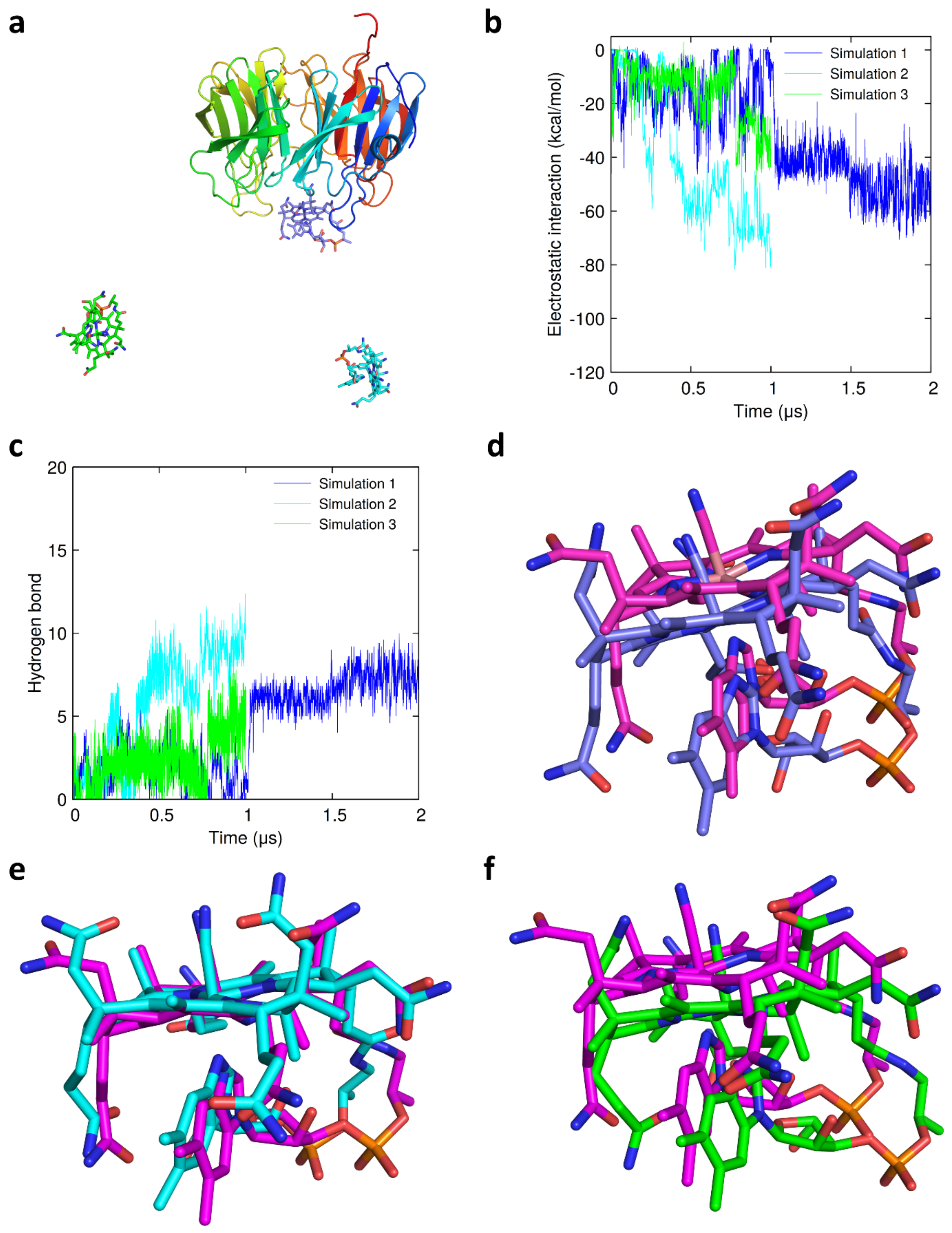

Supplementary Figure 4 l CNCbl acquisition by BtuG2 from different starting locations. a, Representation of the initial positions of CNCbl which are 8.5, 37.1, and 34.9 Å away from the binding pocket for simulation 1 (dark blue), 2 (light blue) and 3 (green), respectively. **b,** Short-range electrostatic interaction energies between the ligand and protein throughout the simulations. **c,** Hydrogen bond interactions between residues of the binding site and CNCbl.

In the crystal structure, 10 hydrogen bonds are present. **d,** Representative overlay of the final ligand position at the end of unbiased simulation 1 (carbon atoms in blue) and the position in the crystal structure (carbon atoms in magenta). **e**, The final position of CNCbl obtained from unbiased simulation 2 (carbon atoms in cyan) compared to the crystal structure (magenta). **f**, Same as **e** but for simulation 3 (carbon atoms in green).

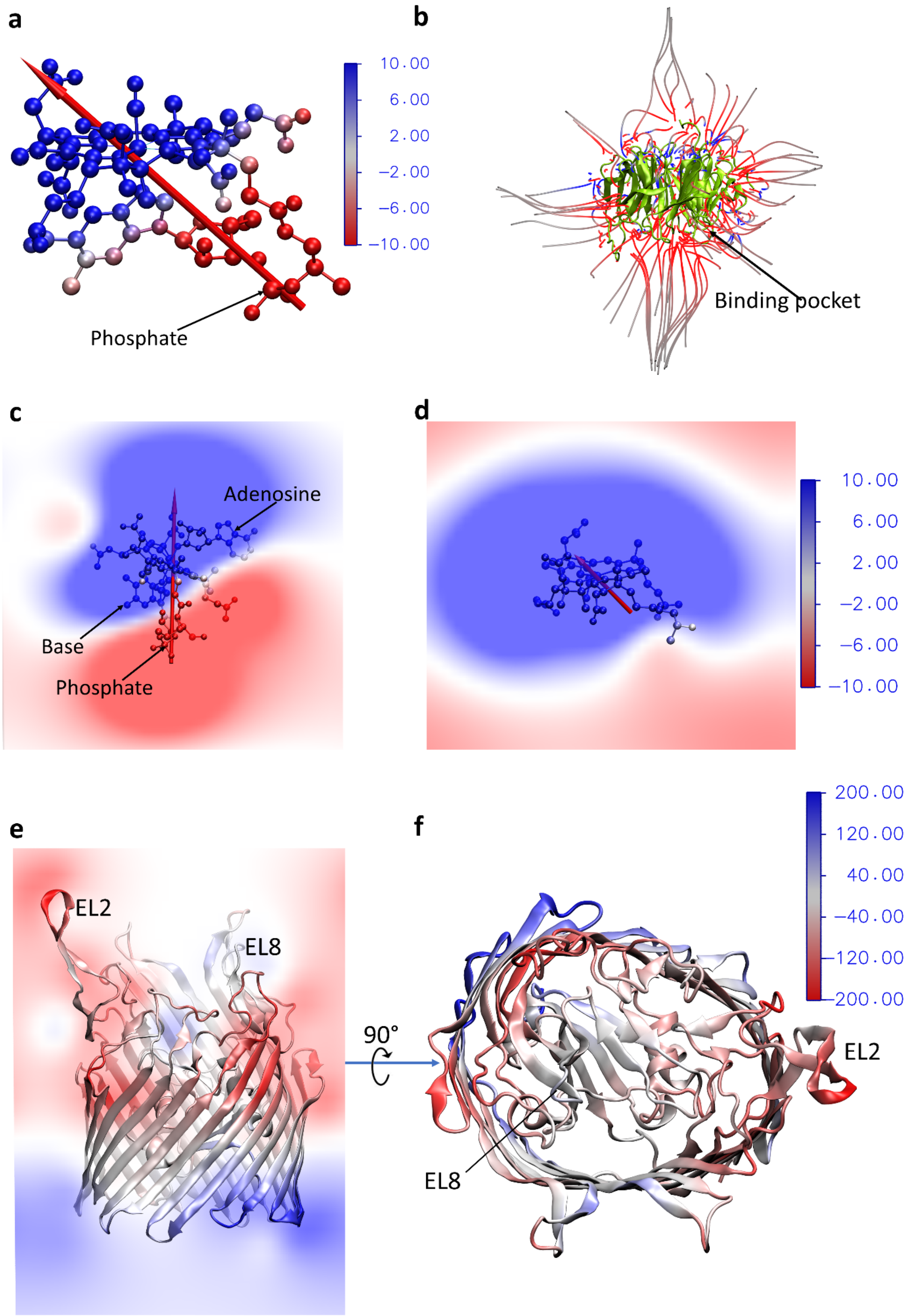
a
b
c
d
e
f
10.00
6.00
2.00
−2.00
−6.00
−10.00
Phosphate
Binding pocket
Adenosine
Base
Phosphate
200.00
120.00
40.00
−40.00
−120.00
−200.00
EL2
EL8
90°

Supplementary Figure 5 l Dipole of CNCbl and Electrostatic potential maps. a, To show the dipole character of CNBl, the electrostatic potential projected onto the molecular structure is shown. The corrin ring is partially positively charged, whereas the nucleotide loop, including the phosphate group, is negatively charged. **b,** Representation of the electric field of BtuG. A large number of electric field lines originate from the binding site of BtuG, caused by an accumulation of negative charges in the protein. The neutral CNCbl has a large dipole moment and is attracted by the binding site. The red field lines point towards the negative charges of the protein and the blue ones towards the positive charges. The line density indicates the strength of the electric field. **c,** Electrostatic potential maps of the neutral AdoCbl molecule with a large dipole moment. The corrin moiety of AdoCbl is partially positively charged whereas the lower ligand is partially negatively charged. **d,** Electrostatic potential maps of the positively charged Cbi. The colour scale is shown from -10, negative surface (red), to 10, positive surface (blue) in units of $k_B T/e$ = 26 mV at 300 K. **e-f,** The electrostatic potential map of BtuB2. The colour scale is shown from -200, negative surface (red), to 200, positive surface (blue).

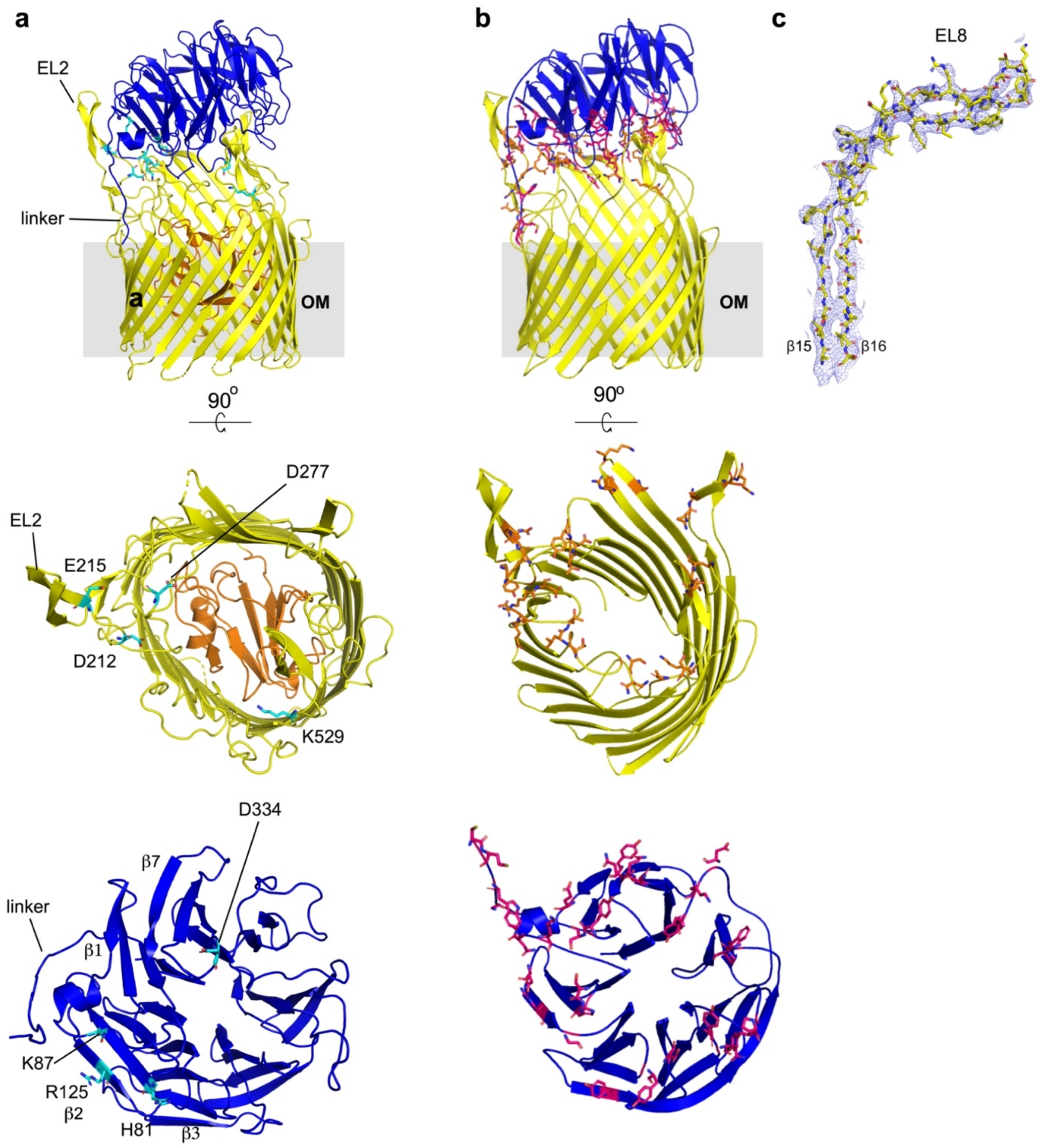

Supplementary Figure 6 l Electrostatic interactions between BtuG2 and BtuB2. a, Cartoon representations showing residues involved in salt bridges in cyan. BtuB2 is in yellow (plug in orange) and BtuG2 in blue. **b**, Residues involved in hydrogen bonding in red for BtuG2 and orange for BtuB2. The bottom panels show the BtuG2 surface viewed from the direction of BtuB2. For clarity, the BtuB2 plug has been removed and loops are smoothened in b. **c**, Figure showing a stick representation of beta strands 15 and 16 and EL8 with the 2Fo-Fc electron density contoured at 1.5 σ.

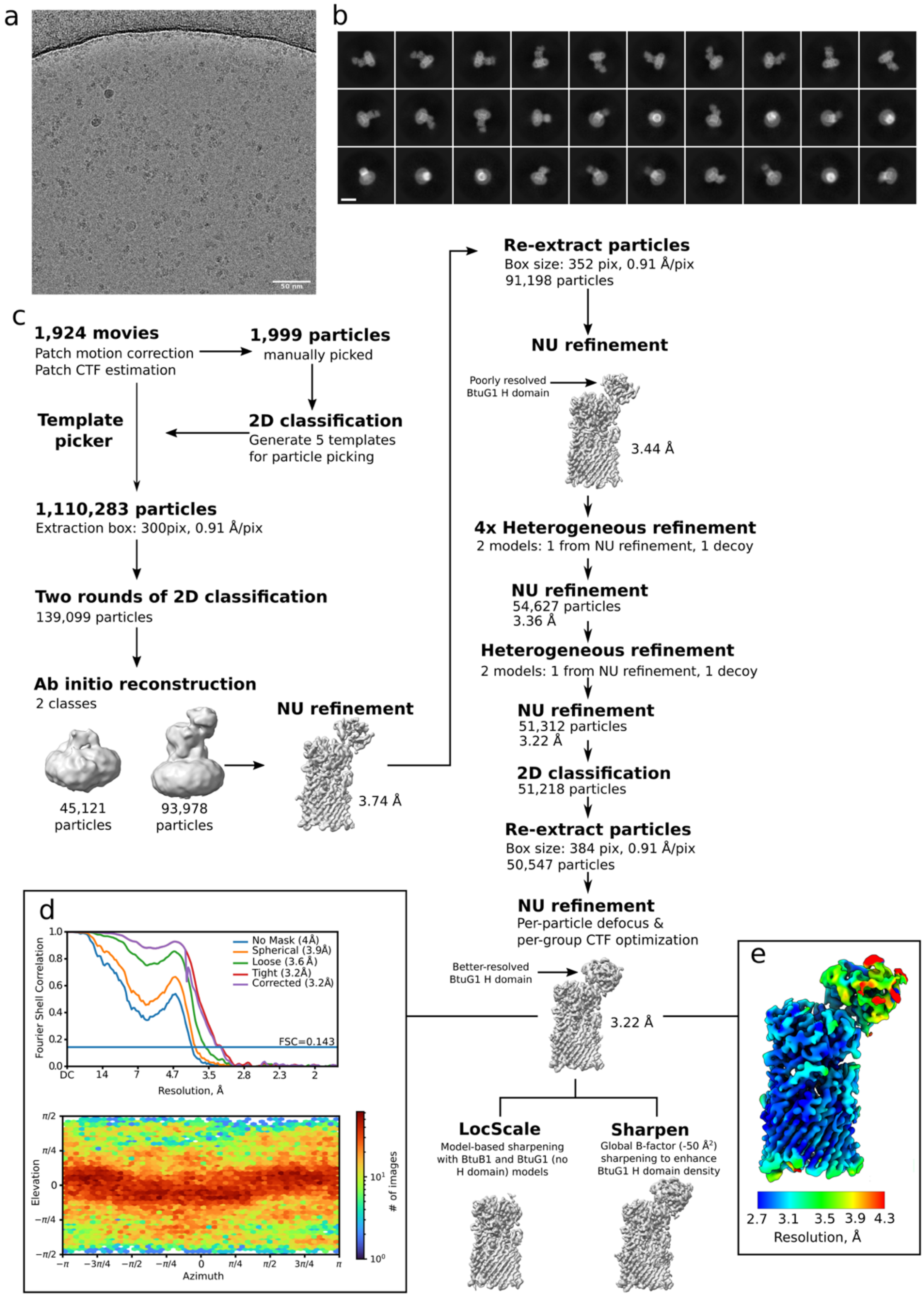

Supplementary Figure 7 l BtuB1G1 cryo-EM data processing. **a**, Representative motion-corrected micrograph out of 1,924 micrographs. **b**, Representative 2D class averages. **c**, Data processing workflow showing steps leading to the final density map. **d**, Fourier shell correlation and particle direction distribution plots for the final reconstruction. **e**, Local resolution plot estimated by cryoSPARC. All resolution estimates are reported using the FSC=0.143 criterion.

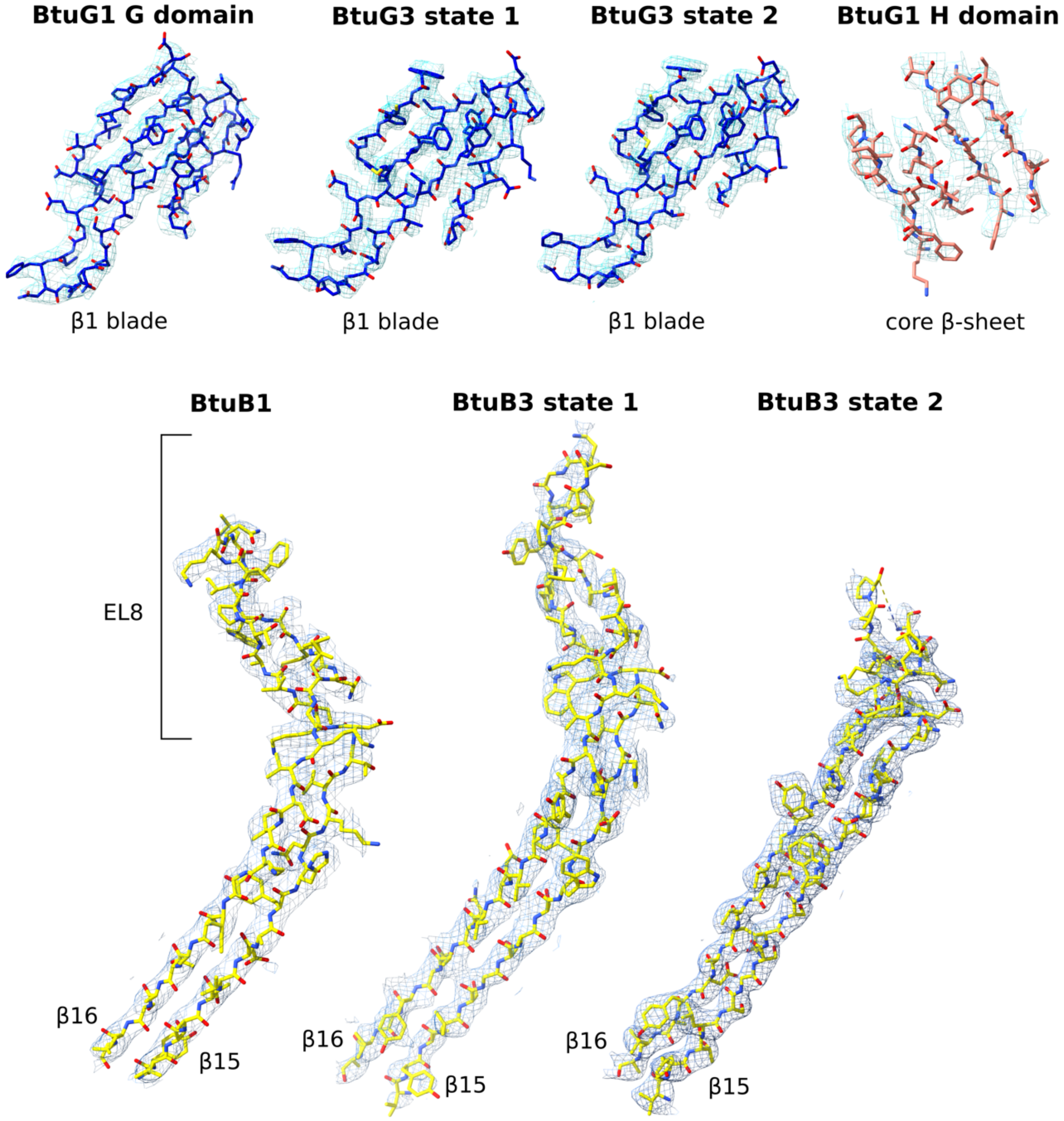

Supplementary Figure 8 l Representative protein model-to-map fits in the cryo-EM structures of BtuB1G1 and BtuB3G3-CNCbl. Electron density for the CNCbl molecule in BtuB3G3 is shown in Supplementary Fig. 11b.

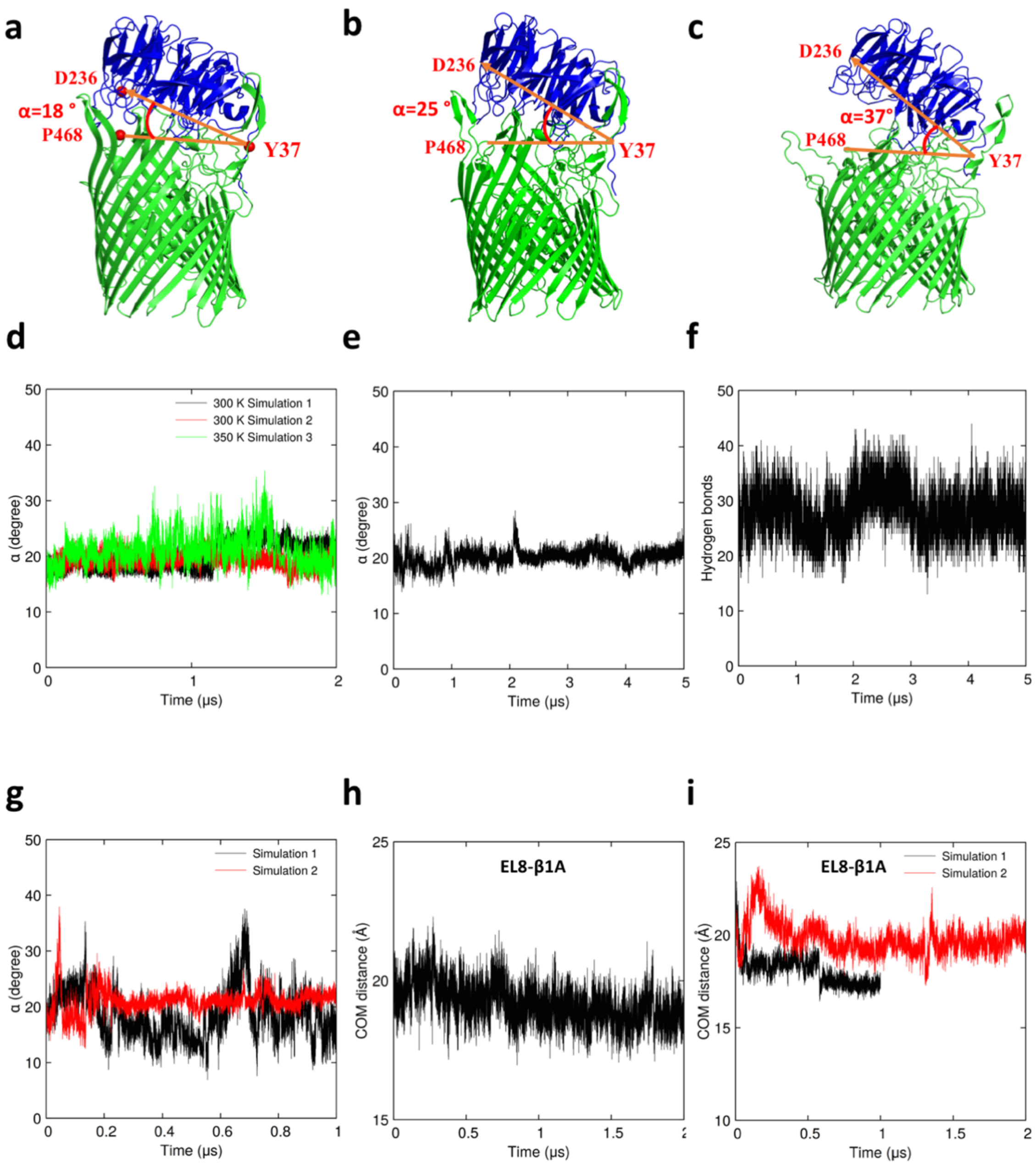

Supplementary Figure 9 | Conformational changes of the BtuB2G2 and BtuB3G3 complexes during unbiased simulations. a, Definition of the opening angle in the crystal structure of BtuB2G2. See text for details. **b,** Unbiased simulations at 300 K reach an opening angle of 25°. **c,** Opening angle of 37° observed at 400 K. **d**, Aperture angle, α, for 2 μs simulations at 300 K and 350 K. The starting point is the closed state seen in the crystal structure (angle α of 18°). **e**, Same as in (**d**) but for the 5 μs simulation. **f**, Average number of hydrogen bonds between BtuB2 and BtuG2 during the 5 μs simulation. **g**, Analysis of the aperture angle for two simulations run at 400K; note the short-duration maximum aperture of around 37° observed in both simulations. **h**, COM distance between EL8 of BtuB3 and βA1 (N54-T64; Supplementary Fig. 3) of BtuG3 during unbiased simulation of BtuB3G3 without CNCbl. The βA1 loop was chosen since it remains stable and is located on the other side of CNCbl relative to EL8. **i**, COM distance between EL8 of BtuB3 and βA1 of BtuG3 during B_{12} translocation. The starting COM distance is ~22 Å.

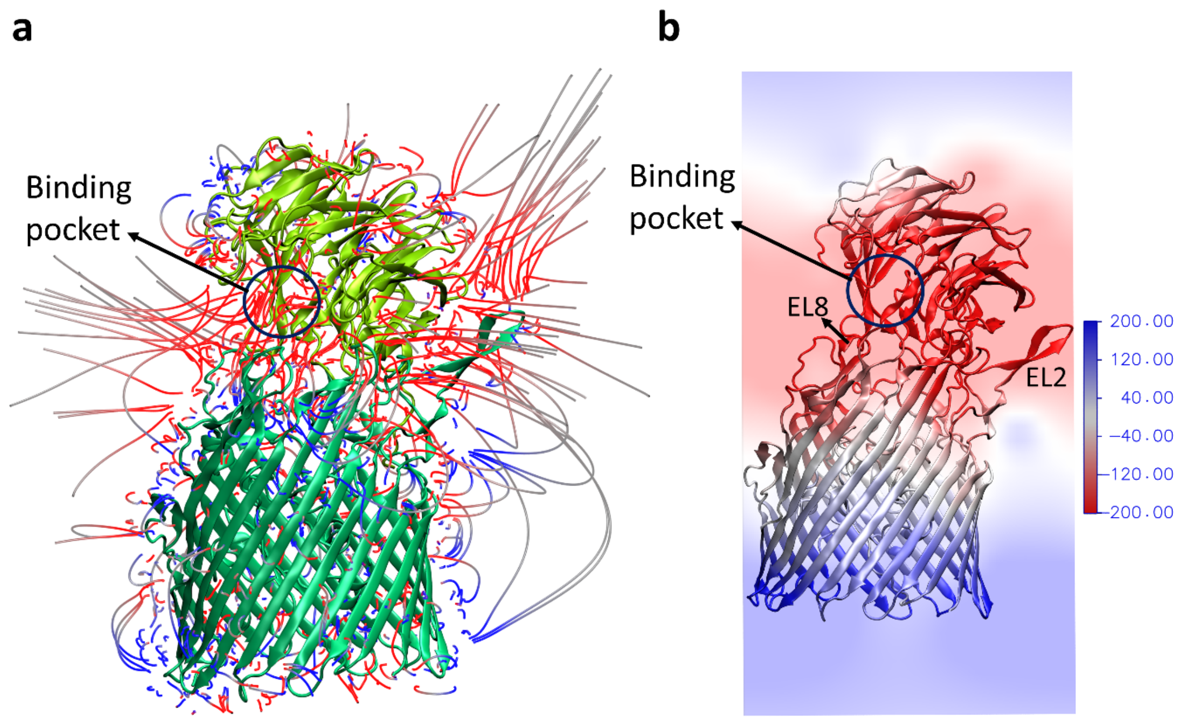

Supplementary Figure 10 l Electrostatics of the partially open BtuB2G2 complex. a, The electric field line calculation of BtuB2G2 with maximum α at 400 K (37°) revealed a significant number of electric field lines originating from the binding site of BtuG2 as was observed for the crystal structure of isolated BtuG2 (Figure 2e). **b,** The electrostatic potential map of BtuB2G2 demonstrates that the BtuG2 binding pocket in BtuB2G2 has a negative electrostatic potential surface similar to the one for isolated BtuG2 (Supplementary Figure 5c-d).

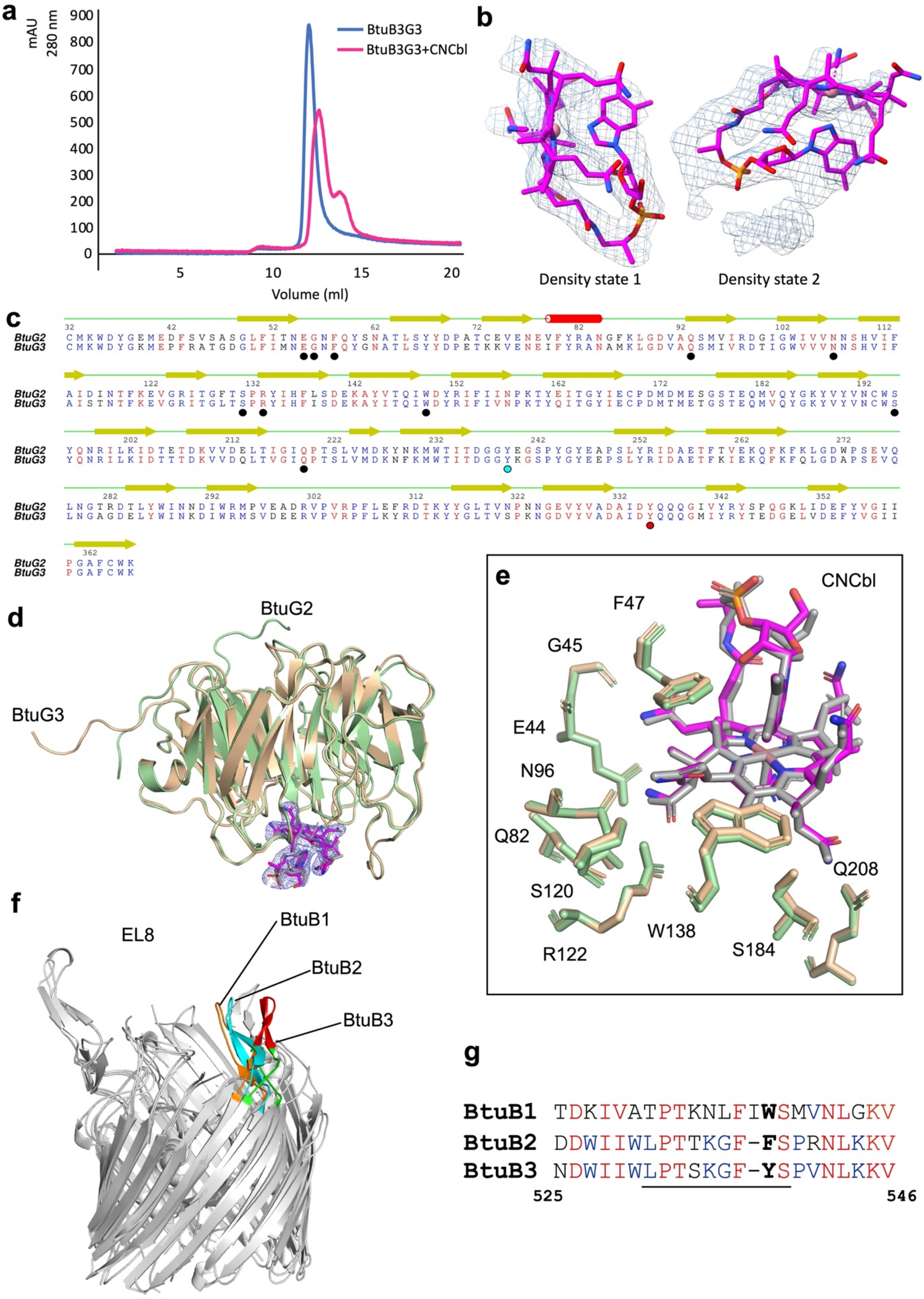

Supplementary Figure 11 l Stability and comparative analysis of BtuB3G3. a, SEC profile for 0.3 mg BtuB3G3 incubated in LDAO buffer at 20°C for 48 hours with (red) and without (blue) 200 molar equivalents of CNCbl. In the presence of the vitamin the profile shows two peaks,

indicating that the complex dissociates. (mAU; milli absorbance unit) **b**, CNCbl modelled for the cryo-EM data state 1 (both panels) with density for state 1 (left panel) and state 2 (right panel), showing the CNCbl displacement in state 2. Both states were superposed prior to making the figures. **c**, Sequence alignment of BtuG2 and BtuG3. Black dots mark the residues involved in CNCbl binding. The red dot represents an additional residue interacting with AdoCbl and the blue dot represents an additional residue interacting with Cbi. **d**, Superposition of the crystal structures of BtuG2-CNCbl (pale green-grey) and BtuG3-CNCbl (wheat-magenta), with a Cα RMSD of 0.4 Å the structures are virtually the same. The 2Fo-Fc electron density at 1.5 σ is shown for CNCbl in the BtuG3-CNCbl crystal structure. **e**, Panel displaying the residues involved in hydrogen bonding between BtuG3/BtuG2 and CNCbl. Colours as in (d). Numbering of the residues corresponds to BtuG3. **f**, Superposition of BtuB1, BtuB2 and BtuB3 state 1 to show the EL8 in orange, blue and green. Red colour indicates the replaced region of the loop for the EL8 mutant of BtuB3. **g**, Sequence alignment of EL8 for BtuB1, BtuB2 and BtuB3. Underlined is the replaced region; in bold is the aromatic residue that forms a hydrogen bond with CNCbl in BtuB3 and which is conserved in BtuB2 and BtuB1.

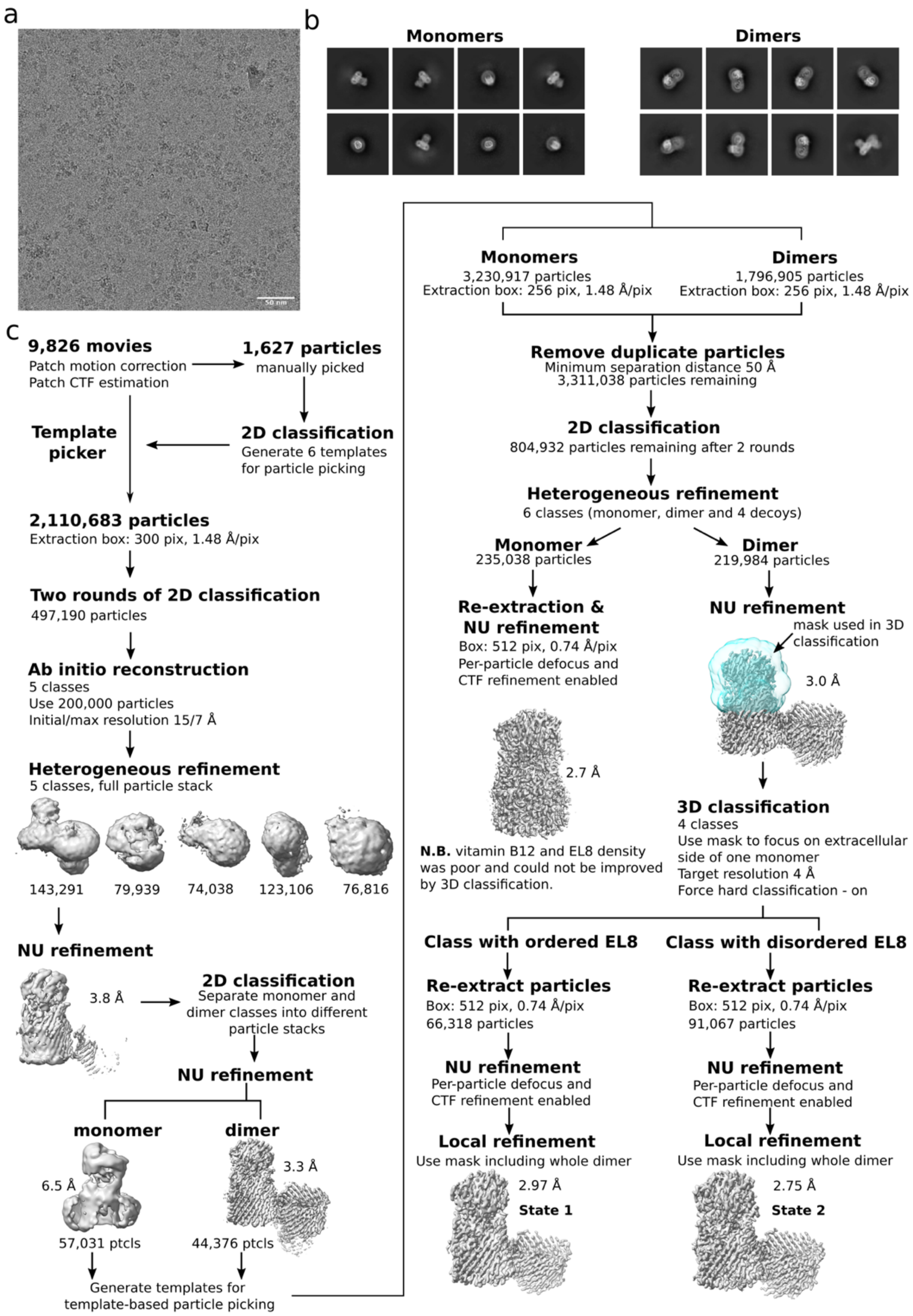

Supplementary Figure 12 l BtuB3G3-CNCbl cryo-EM data processing. **a**, Representative motion-corrected micrograph out of 9,826 micrographs. **b**, Representative 2D class averages for BtuB3G3 monomeric and dimeric complexes. **c**, Data processing workflow showing steps leading to the final density maps with ordered (state 1) and disordered EL8 (state 2). All resolution estimates are reported using the FSC=0.143 criterion. Fourier shell correlation and particle direction distribution plots are shown in Supplementary Figure 13.

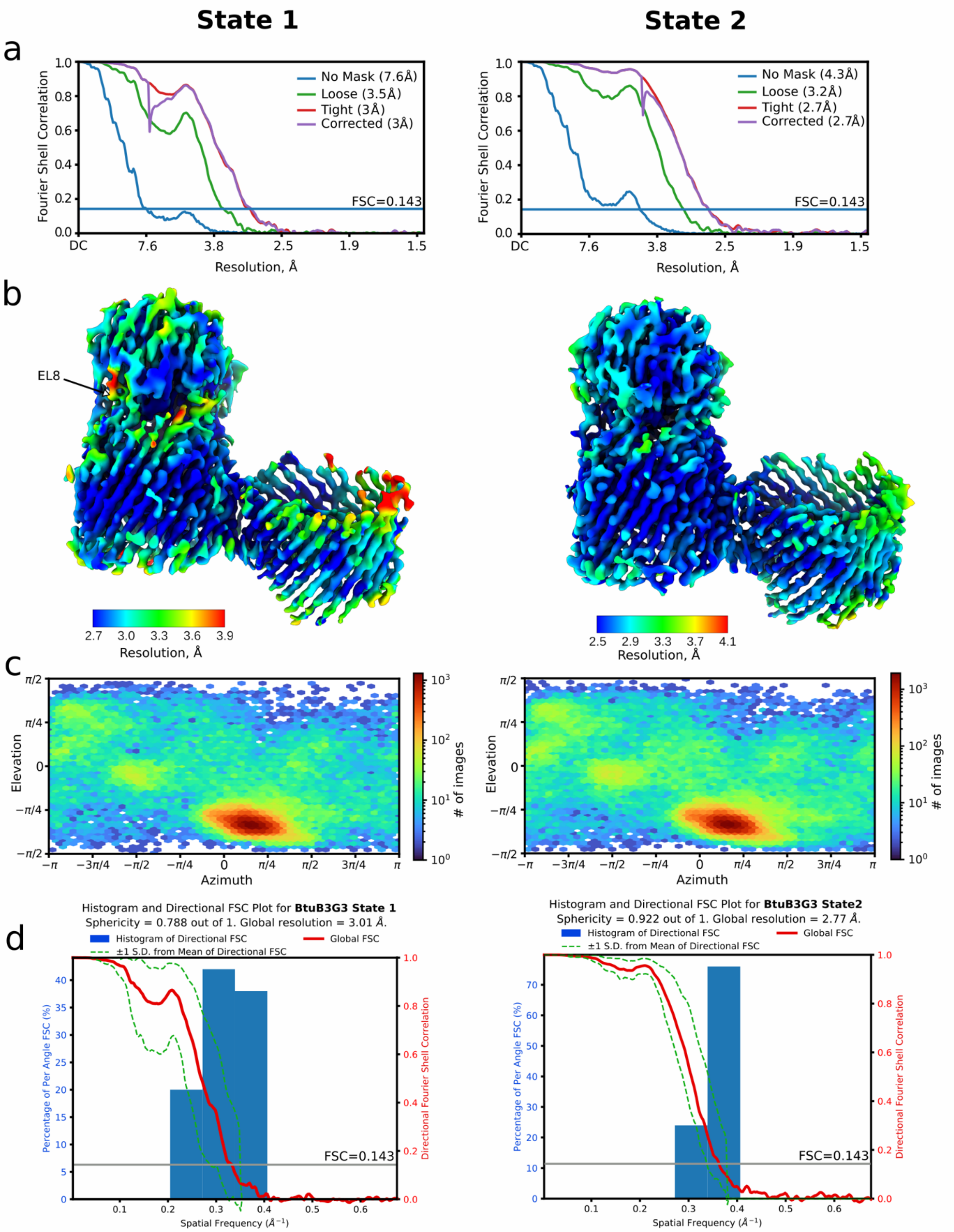

Supplementary Figure 13 l BtuB3G3 cryo-EM final density map properties. a, Fourier shell correlation curves. **b**, Local resolution estimates. **c-d**, Particle direction distribution plots showing preferential orientation in (**c**) and 3DFSC plots[1] in (**d**) for BtuB3G3 state 1 (left column) and state 2 (right column).

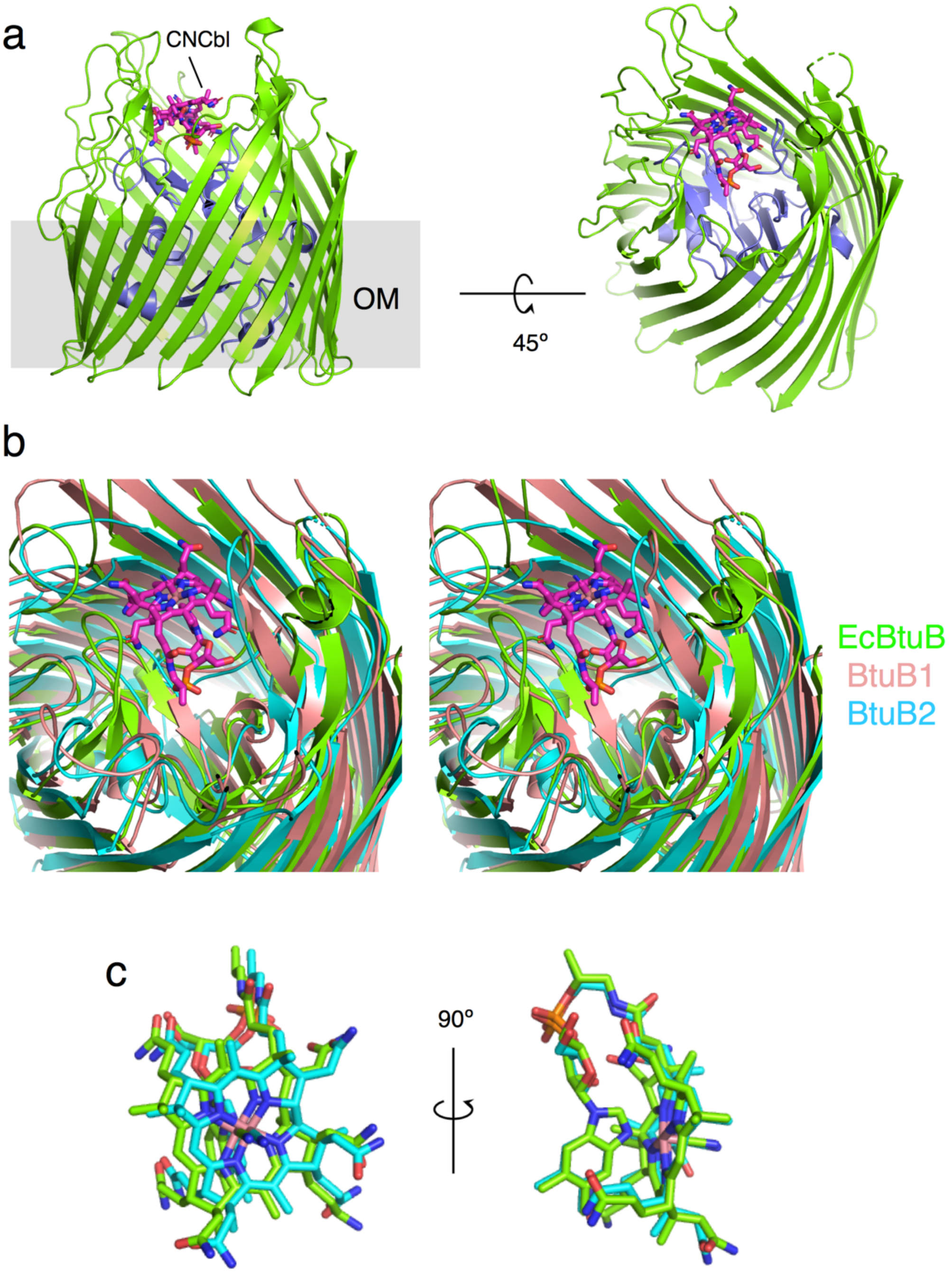

Supplementary Figure 14 | Comparison of B_{12} binding sites in different BtuBs. a, Cartoon model of EcBtuB viewed from the OM plane (left) and from the outside of the cell, with bound CNCbl shown as a stick model (PDB ID 2GSK)[2] . The EcBtuB plug is slate blue. **b**, Stereo cartoon superpositions of EcBtuB and *B. theta* BtuB1 and BtuB2, showing the similarity of the B_{12} binding sites. Loops have been smoothened for clarity. **c**, Comparison of bound CNCbl in EcBtuB with docked CNCbl in *B. theta* BtuB2. The views were generated via superposition of the BtuB proteins.

Supplementary References

1. Tan, Y. Z. *et al.* Addressing preferred specimen orientation in single-particle cryo-EM through tilting. *Nat Methods* 14, 793–796 (2017).

2. Shultis, D. D., Purdy, M. D., Banchs, C. N. & Wiener, M. C. Outer Membrane Active Transport: Structure of the BtuB:TonB Complex. *Science* 312, 1396–1399 (2006).
