# Supplementary material for: BtuB TonB-dependent transporters and BtuG surface lipoproteins form stable complexes for vitamin B12 uptake in gut Bacteroides
Source: Nat Commun. 2023 Aug 5;14:4714. doi: 10.1038/s41467-023-40427-2 (PMC10404256; doi:10.1038/s41467-023-40427-2)
Supplement: Supplementary file 3 — Description of Additional Supplementary Files [file 41467_2023_40427_MOESM3_ESM.pdf]

Description of Additional Supplementary Files

Supplementary Movie 1: Binding of CNCbl by BtuG2. Molecular dynamic simulation showing how CNCbl binds BtuG2 in an association-dissociation fashion. The β5A loop is represented in red.

Supplementary Movie 2: Translocation of CNCbl by BtuB3G3. Video showing a 1 μs-long unbiased MD simulation in which a CNCbl translocation event can be observed. The CNCbl translocation event likely happens via a pushing mechanism which arose from the repulsive force between loop EL8 and CNCbl. Loop β5A of BtuG3 is represented in red and EL8 of BtuB3 in magenta. BtuG3 and BtuB3 are depicted in blue and green, respectively.
