# Supplementary material for: BtuB TonB-dependent transporters and BtuG surface lipoproteins form stable complexes for vitamin B12 uptake in gut Bacteroides
Source: Nat Commun. 2023 Aug 5;14:4714. doi: 10.1038/s41467-023-40427-2 (PMC10404256; doi:10.1038/s41467-023-40427-2)
Supplement: Supplementary file 6 — Reporting Summary [file 41467_2023_40427_MOESM6_ESM.pdf]

nature portfolio

Corresponding author(s): Bert van den Berg
Ulrich Kleinekathöfer

Last updated by author(s): 19 July 2023

Reporting Summary

Nature Portfolio wishes to improve the reproducibility of the work that we publish. This form provides structure for consistency and transparency in reporting. For further information on Nature Portfolio policies, see our Editorial Policies and the Editorial Policy Checklist.

Statistics

For all statistical analyses, confirm that the following items are present in the figure legend, table legend, main text, or Methods section.

n/a	Confirmed	
☐	☒	The exact sample size (*n*) for each experimental group/condition, given as a discrete number and unit of measurement
☐	☒	A statement on whether measurements were taken from distinct samples or whether the same sample was measured repeatedly
☐	☒	The statistical test(s) used AND whether they are one- or two-sided<br>*Only common tests should be described solely by name; describe more complex techniques in the Methods section.*
☒	☐	A description of all covariates tested
☐	☒	A description of any assumptions or corrections, such as tests of normality and adjustment for multiple comparisons
☐	☒	A full description of the statistical parameters including central tendency (e.g. means) or other basic estimates (e.g. regression coefficient) AND variation (e.g. standard deviation) or associated estimates of uncertainty (e.g. confidence intervals)
☐	☒	For null hypothesis testing, the test statistic (e.g. *F*, *t*, *r*) with confidence intervals, effect sizes, degrees of freedom and *P* value noted<br>*Give P values as exact values whenever suitable.*
☒	☐	For Bayesian analysis, information on the choice of priors and Markov chain Monte Carlo settings
☒	☐	For hierarchical and complex designs, identification of the appropriate level for tests and full reporting of outcomes
☒	☐	Estimates of effect sizes (e.g. Cohen's *d*, Pearson's *r*), indicating how they were calculated

Our web collection on statistics for biologists contains articles on many of the points above.

Software and code

Policy information about availability of computer code

Data collection	Mass spectrometry for proteomics: Xcalibur 4.4 (ThermoFisher Scientific)<br><br>Growth curves: Biotek Gen5 (2.09 Agilent)<br><br>CryoEM data collection: EPU v3.2
Data analysis	Growth curves were analysed and plotted using the R package (Version 4.2.3) Ggplot2 (Version 3.4.2)<br><br>Proteomics: MaxQuant V 2.0.3.0 , Perseus 2.0.3.1, Limma package in R package (Version 4.2.3) Ggplot2 (Version 3.4.2)<br><br>X-ray data processing, modeling, and refinement: XDS (Feb 5, 2021), Pointless (1.12.14), Aimless (0.7.9),Dials (3.11.1), Refmac (5.8.0267), Phenix (1.20.1-4487, includes: autosolve, autobuild, phenix refine, phaser and sculptor), coot (0.9.8.8) and MolProbity (4.2).<br><br>For CryoEM dat analysis and structure refinement: cryoSPARC v3.3.2, cryoSPARC v4.1.2, CCPEM v1.6 - program Buccaneer, Coot v0.9.8.8, AlphaFold v2, Phenix v1.20.1-4487 and UCSF ChimeraX v1.5<br><br>Software used for molecular dynamics: CHARMM-GUI membrane builder. PROPKA webserver, GROMACS molecular dynamics software (5.1.14), CHARMM36-m forcefield and MODELLER (10.4).

For manuscripts utilizing custom algorithms or software that are central to the research but not yet described in published literature, software must be made available to editors and reviewers. We strongly encourage code deposition in a community repository (e.g. GitHub). See the Nature Portfolio guidelines for submitting code & software for further information.

Data

Policy information about availability of data

All manuscripts must include a data availability statement. This statement should provide the following information, where applicable:

- Accession codes, unique identifiers, or web links for publicly available datasets
- A description of any restrictions on data availability
- For clinical datasets or third party data, please ensure that the statement adheres to our policy

The mass spectrometry proteomics data have been deposited to the ProteomeXchange Consortium via the PRIDE partner repository with the data set identifier: PXD038230 (https://proteomecentral.proteomexchange.org/cgi/GetDataset?ID=PXD038230). For X-ray structures coordinates and structure factors have been deposited in the Protein Data Bank with accession codes 8BMX for BtuG2-CNCbl (https://www.wwpdb.org/pdb?id=pdb_00008bmx), 8BMY for BtuG2-AdoCbl (https://www.wwpdb.org/pdb?id=pdb_00008bmy), 8BZM (https://www.wwpdb.org/pdb?id=pdb_00008bzm) for BtuG2-Cbi, 8BM0 for BtuB2G2 (https://www.wwpdb.org/pdb?id=pdb_00008bm0) and 8OKV for BtuG3-CNCbl (https://www.wwpdb.org/pdb?id=pdb_00008okv). EM structure coordinates have been deposited in the Protein Data Bank and EM maps in the Electron Microscopy Data Bank with accession codes 8BLW (https://www.wwpdb.org/pdb?id=pdb_00008blw) and EMD-16114 (https://www.ebi.ac.uk/emdb/EMD-16114) for BtuB1G1, 8P98 (https://www.wwpdb.org/pdb?id=pdb_00008p98) and EMD-17575 (https://www.ebi.ac.uk/emdb/EMD-17575) for BtuB3G3-CNCbl state1 and 8P97 (https://www.wwpdb.org/pdb?id=pdb_00008p97) and EMD-17574 (https://www.ebi.ac.uk/emdb/EMD-17574) for BtuB3G3-CNCbl state2. The pdb model used for Molecular replacement was previously deposited as 3DSM (https://www.wwpdb.org/pdb?id=pdb_00003dsm). Initial and final conformations for the molecular dynamics analysis have been deposited in the Zenodo repository (https://doi.org/10.5281/zenodo.8164805)

Human research participants

Policy information about studies involving human research participants and Sex and Gender in Research.

Reporting on sex and gender	N/A
Population characteristics	N/A
Recruitment	N/A
Ethics oversight	N/A

Note that full information on the approval of the study protocol must also be provided in the manuscript.

Field-specific reporting

Please select the one below that is the best fit for your research. If you are not sure, read the appropriate sections before making your selection.

☒ Life sciences ☐ Behavioural & social sciences ☐ Ecological, evolutionary & environmental sciences

For a reference copy of the document with all sections, see nature.com/documents/nr-reporting-summary-flat.pdf

Life sciences study design

All studies must disclose on these points even when the disclosure is negative.

Sample size	Proteomics: Five biological replicates were performed for each condition, this is typical for a proteomics experiment where modest true effects are expected (such as in this case of comparing the impact of high vs. low cyanacobalamin) and statistical tests such as the t-test are used. See DOI 10.1007/978-1-0716-1024-4_1 for further details.
Data exclusions	Proteomics: This section is not applicable; no mass spectrometry raw data files were excluded from proteomics analysis.
Replication	Proteomics experiments: The pipeline for data processing included steps at which reproducibility within and between conditions was checked. This included checking that LFQ intensity of proteins was similar in each sample, and a clustering (principal component) analysis. These confirmed replicates were similar.
Randomization	The aim was to compare differences between high and low concentrations of cyanocobalamin, hence randomization was not needed.
Blinding	The aim was to compare differences between high and low concentrations of cyanocobalamin, hence blinding was not needed.

Reporting for specific materials, systems and methods

We require information from authors about some types of materials, experimental systems and methods used in many studies. Here, indicate whether each material, system or method listed is relevant to your study. If you are not sure if a list item applies to your research, read the appropriate section before selecting a response.

Materials & experimental systems

n/a	Involved in the study
☒	☐ Antibodies
☒	☐ Eukaryotic cell lines
☒	☐ Palaeontology and archaeology
☒	☐ Animals and other organisms
☒	☐ Clinical data
☒	☐ Dual use research of concern

Methods

n/a	Involved in the study
☒	☐ ChIP-seq
☒	☐ Flow cytometry
☒	☐ MRI-based neuroimaging
