# Supplementary material for: BtuB TonB-dependent transporters and BtuG surface lipoproteins form stable complexes for vitamin B12 uptake in gut Bacteroides
Source: Nat Commun. 2023 Aug 5;14:4714. doi: 10.1038/s41467-023-40427-2 (PMC10404256; doi:10.1038/s41467-023-40427-2)

Uncropped gel Figure 1c

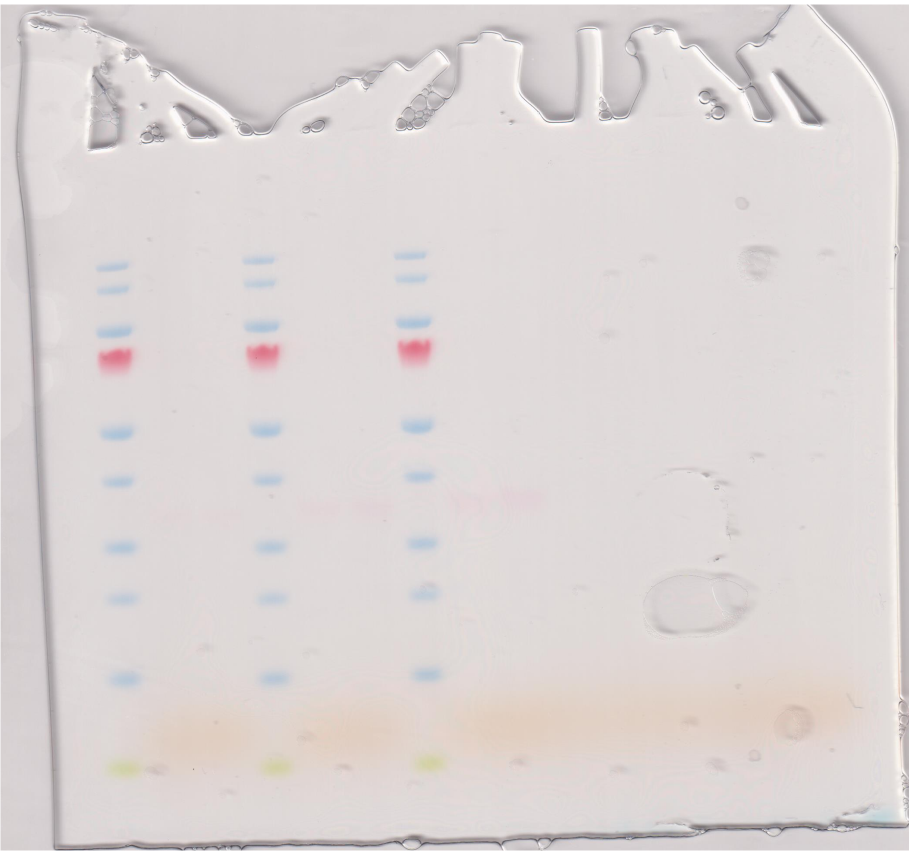

Uncropped gel Figure 1c

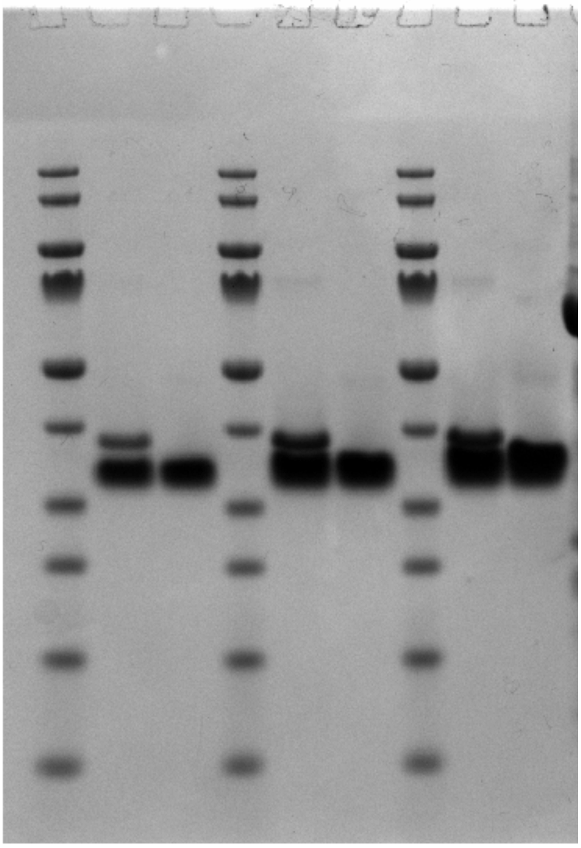

Uncropped gel Figure 3a

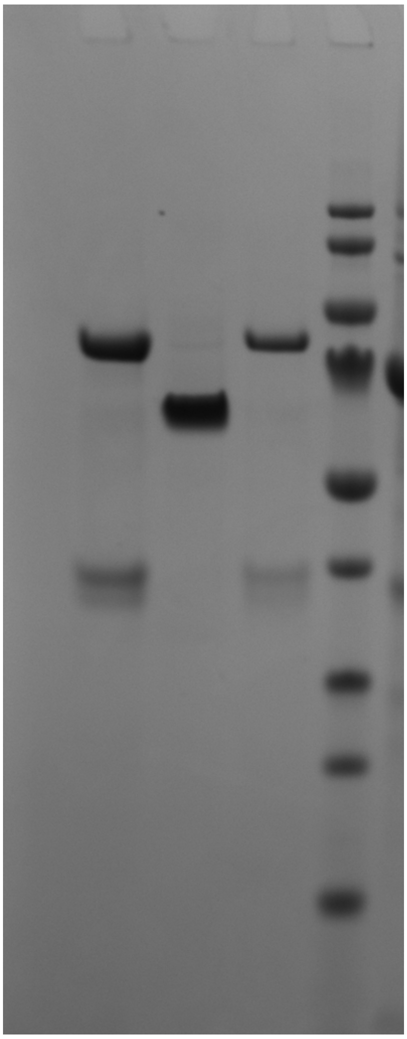

Uncropped gel Figure 6g

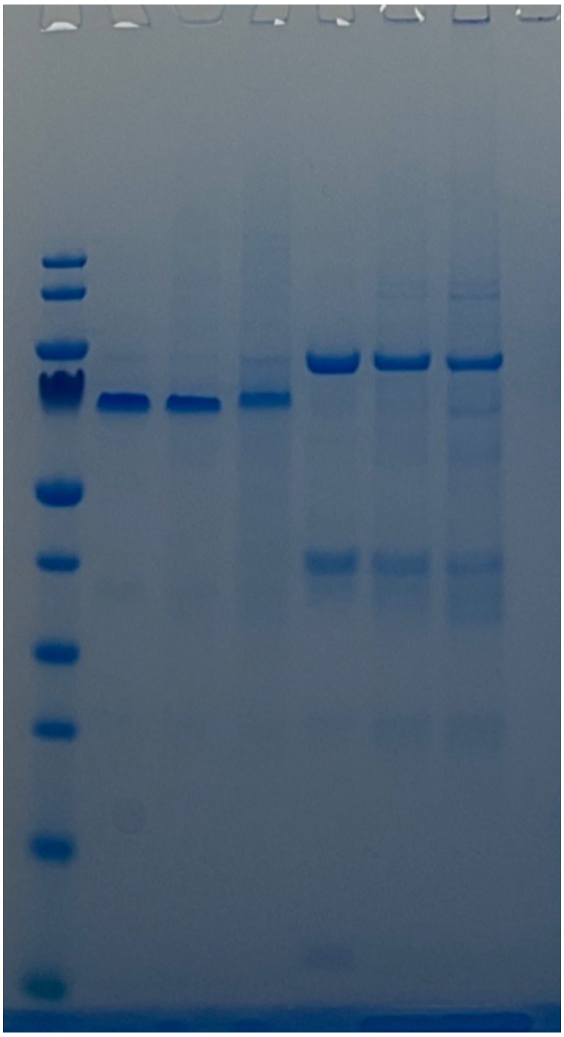

Uncropped gel supplementary Figure 1a

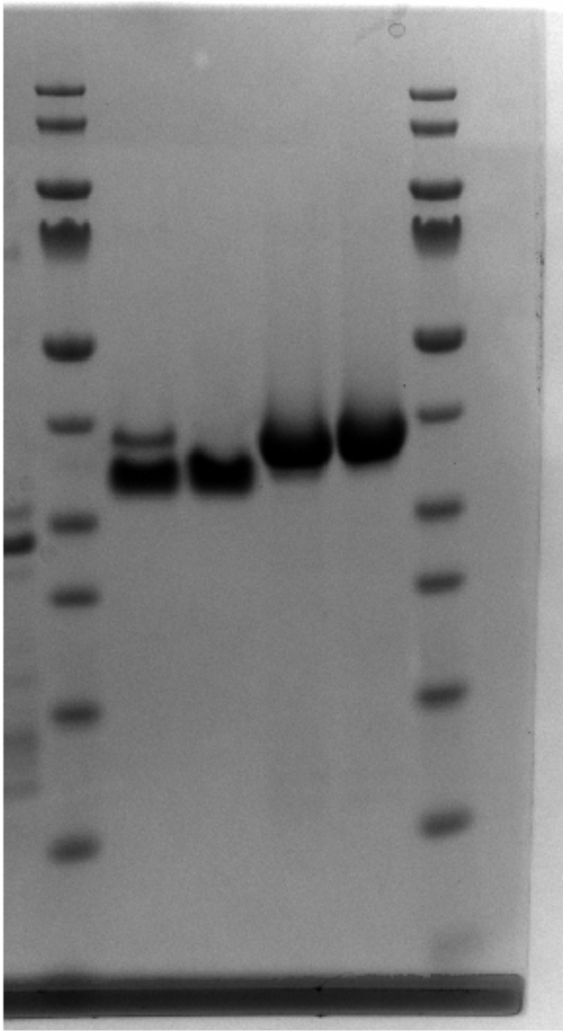

Supplement: Supplementary file 7 — Source Data [file 41467_2023_40427_MOESM7_ESM.zip › Source Data/Source Data uncropped gels.pdf]
