# Supplementary figures and images for: BtuB TonB-dependent transporters and BtuG surface lipoproteins form stable complexes for vitamin B12 uptake in gut Bacteroides
Source: Nat Commun. 2023 Aug 5;14:4714. doi: 10.1038/s41467-023-40427-2 (PMC10404256; doi:10.1038/s41467-023-40427-2)

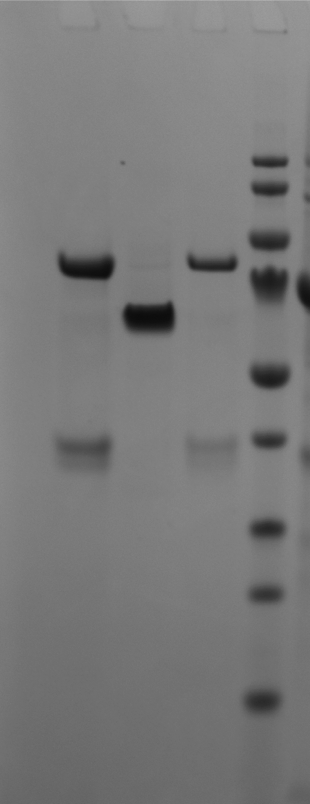

Supplement: Supplementary file 7 — Source Data [file 41467_2023_40427_MOESM7_ESM.zip › Source Data/F3a.png]

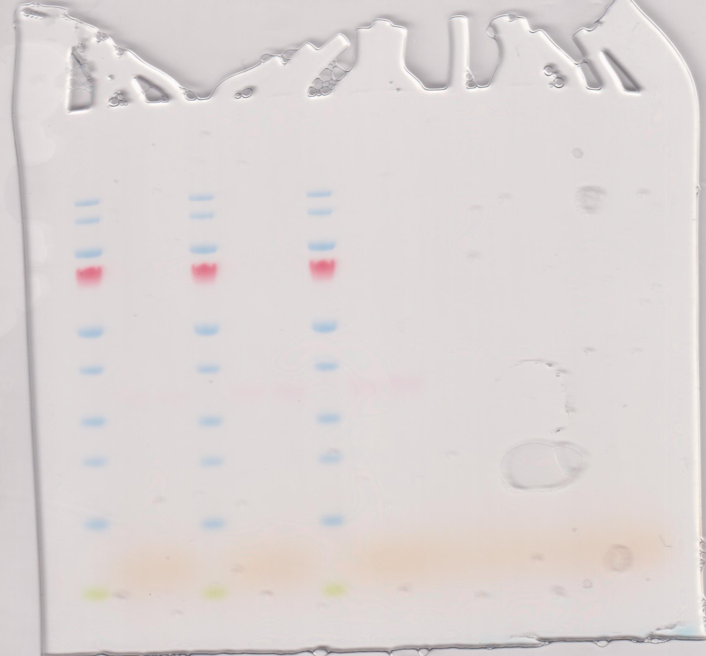

Supplement: Supplementary file 7 — Source Data [file 41467_2023_40427_MOESM7_ESM.zip › Source Data/F1c.png]

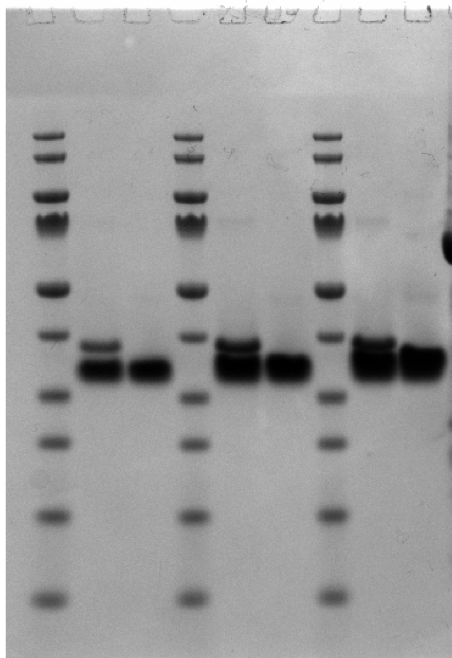

Supplement: Supplementary file 7 — Source Data [file 41467_2023_40427_MOESM7_ESM.zip › Source Data/F1cbis.png]

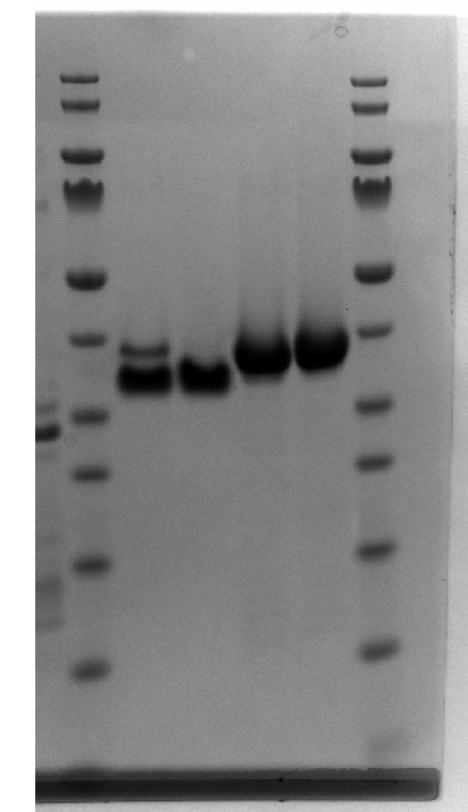

Supplement: Supplementary file 7 — Source Data [file 41467_2023_40427_MOESM7_ESM.zip › Source Data/SupF1a.png]

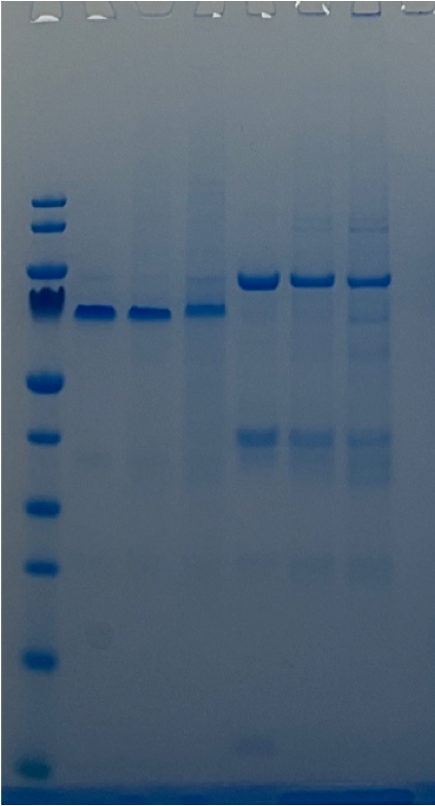

Supplement: Supplementary file 7 — Source Data [file 41467_2023_40427_MOESM7_ESM.zip › Source Data/F6g.png]
